# Impact of Change, Fluctuation, or Variability in Weight on the Risk of Nonalcoholic Fatty Liver Disease in General Population: A Systematic Review and Meta‐Analysis

**DOI:** 10.1002/hsr2.71255

**Published:** 2025-09-29

**Authors:** Dorsa Alijanzadeh, Masoud Noroozi, Negin Rostami, Narges Norouzkhani, Mahdie ShojaeiBaghini, Saeed Zivari Lashkajani, Ali Mirzaei, Maryam Dianati, Maryam Salimi, Hamidreza Sadeghsalehi, Faezeh Jadidian, Sajjad Ghane Ezabadi, Mohammad Sadegh Fallahi, Mobina Fathi, Alaleh Alizadeh, Mahsa Asadi Anar, Niloofar Deravi

**Affiliations:** ^1^ School of Medicine Shahid Beheshti University of Medical Sciences Tehran Iran; ^2^ Department of Biomedical Engineering, Faculty of Engineering University of Isfahan Isfahan Iran; ^3^ Department of Medical Islamic Azad Univeristy of Mashhad Mashhad Iran; ^4^ Department of Medical Informatics, Faculty of Medicine Mashhad University of Medical Sciences Mashhad Iran; ^5^ Medical Informatics Research Center, Institute for Futures Studies in Health Kerman University of Medical Sciences Kerman Iran; ^6^ Kashan University of Medical Sciences Kashan Iran; ^7^ Mashhad University of Medical Sciences Mashhad Iran; ^8^ Rafsanjan University of Medical Sciences Rafsanjan Iran; ^9^ Department of Pharmacy Zanjan University of Medical Sciences Zanjan Iran; ^10^ Department of Artificial Intelligence in Medical Sciences Iran University Of Medical Sciences Tehran Iran; ^11^ Multiple Sclerosis Research Center, Neuroscience Institute Tehran University of Medical Sciences Tehran Iran; ^12^ School of Medicine Tehran University of Medical Sciences Tehran Iran; ^13^ Faculty of Medicine, Mashhad Branch Islamic Azad University Mashhad Iran

**Keywords:** body weight changes, NAFLD, nonalcoholic fatty liver disease, obesity

## Abstract

**Background and Aim:**

Numerous studies on the impact of weight changes on nonalcoholic fatty liver disease are being conducted; therefore, this systematic review aims to critically discuss the impact of change, fluctuation, or variability in weight on the risk of nonalcoholic fatty liver disease in the general population.

**Methods:**

Data from three databases, PubMed/Medline, Google Scholar, and Scopus, which were available until June 2024, were used to compile the materials for our research. Only English‐language publications were submitted for this study, and they were evaluated based on their titles, abstracts, and complete texts and.

**Result:**

Eight studies (three cross‐sectional articles and five cohort articles) involving 147,601 participants from Japan, China, Korea, and the United States were included. The results of this study showed that weight loss had significantly increased odds of developing NAFLD independently and per 1 kg compared to the stable weight control group. (OR = 1.16, 95% CI: 1.118–1.208, *p* < 0.0001, *I* ^2^ = 86%, OR = 1.186, 95% CI: 1.142–1.230, *p* < 0.0001, *I* ^2^ = 89.2%). The findings of seven articles indicated that the odds of experiencing NAFLD among patients with increased weight was 0.697 (OR = 0.697, 95% CI: 0.391–1.002, *p* < 0.0001, *I* ^2^ = 99.9%), suggesting a slightly reduced likelihood compared to the control group. The sensitivity analysis supported the robustness of the findings.

**Conclusion:**

Nonalcoholic fatty liver disease is increasing in industrialized nations and has a significant financial impact on people′s health and healthcare systems. It can be controlled by emphasis on reducing weight change and maintaining a healthy weight.

## 1 Introduction

Reaching epidemic status, nonalcoholic fatty liver disease (NAFLD) has become a severe health concern. NAFLD, with diverse manifestations ranging from simple hepatic steatosis to steatohepatitis, is the primary cause of liver disease [1]. NAFLD is strongly associated with an increased risk of complications, such as diabetes mellitus, cancer, and cardiovascular diseases [2, 3, 4, 5]. Obesity is a fundamental risk factor of NAFLD [6]. Obesity has also reached pandemic levels in recent years [7]. It was shown that adverse consequences of obesity are considerably associated with NAFLD [8]. Obesity itself facilitates the occurrence of other impairments, such as type 2 diabetes and insulin resistance, totally increases the risk of metabolic syndrome [9, 10, 11], and NAFLD is known as a hepatic manifestation of metabolic syndrome [12].

There are obese individuals without any metabolic abnormalities. Metabolically healthy obese (MHO) individuals are not exempt from an increased risk of NAFLD [13], liver fibrosis [14], or all‐cause mortality [15]. As NAFLD develops, MHO becomes metabolically unhealthy [16, 17], leading to a higher risk of metabolic syndrome [18], diabetes [19], and cardiovascular disease [20]. Moreover, lean subjects are at a higher risk of NAFLD [21, 22].

Despite the absence of specific treatments for NAFLD, NAFLD is reversible [6]. Several studies have shown that weight loss improves hepatic steatosis and relevant metabolic indices [23]. Therefore, it has become an established therapeutic strategy for the reduction of obesity‐related metabolic risk factors and NAFLD in a dose‐dependent manner [24]. Weight loss of only approximately 7%–10% has a significant impact on overweight and obese people who suffer from nonalcoholic steatohepatitis in terms of reduction in liver fat content, attenuation of liver inflammation, and amelioration of fibrosis [24]. Even in lean subjects with average weight, NFLAD declined, and steatosis improved upon weight loss [25]. Therefore, the primary goal of NAFLD treatment is weight reduction, which has been further documented in clinical guidelines as a typical method of NAFLD management [26].

However, most subjects fail to achieve or maintain a healthy weight, which imposes repeated rounds of weight loss and regains [8]. This condition, called weight change, fluctuation, or cycling, increases the risk of future cardiovascular outcomes and death [27, 28, 29]. To substantiate, this weight regain has been reported in most NAFLD patients who experience weight loss [30]. One study reported that about four‐fifths of subjects with weight loss (more than 10%) regain it within 1 year [31]. Such weight change puts those with coronary artery disease at a higher risk of diabetes mellitus [32] or cardiovascular events and death [28]. Indeed, the general population is endangered because of the incidence of type 2 diabetes following weight change [33, 34].

Although lifestyle modification, which is routinely represented in weight loss, is assumed to be the frontline strategy against NAFLD [35, 36], the effects of weight fluctuations in reducing the risk of NAFLD remain to be elucidated. Currently, one of the exciting area of research is the sequelae of weight fluctuations on health status [32, 33], specifically concerning the risk of NAFLD. Therefore, the effects of weight change on the risk of NAFLD should be scrutinized to identify efficient preventive and therapeutic approaches. Due to the paucity of data, this systematic review investigated the association of weight change, fluctuation, and variability with the risk of NAFLD, which was previously neglected to the best of our knowledge.

## 2 Pathophysiology of NAFLD

Nutritional, hormonal, and genetic factors are involved in the pathogenesis of this systemic disease, with complex interactions [37]. Understanding the disease′s association and pathophysiological features is crucial for understanding the risk factors and treating the condition. Therefore, it can be helpful for clinical purposes as well. The complex pathogenesis of NAFLD mirrors its management. Therefore, special attention must be paid to the pathophysiology [38].

A significant proportion of patients with NAFLD are obese. A state of activation in the dopamine and opioid receptors and increased cerebral blood flow in areas responsible for motivation and reward are reported to contribute to obesity in patients with NAFLD [37, 39]. In addition to the above‐mentioned pathway, increased levels of gut‐derived hormones that stimulate hunger, including ghrelin, are related to an increase in circulating triglycerides [40]. Furthermore, leptin primarily reduces food intake and increases energy levels [37].

In terms of nutrition, people with higher weight have a high‐fructose diet and high soda intake. These nutritional factors can cause chronic inflammation, which is an essential component of the disease pathophysiology in patients with obesity [37]. Interestingly, patients with nonalcoholic steatohepatitis have been shown to possess disrupted gut epithelial tight junctions. This leads to gastrointestinal bacteria having access to the systemic circulation, releasing pro‐inflammatory cytokines, and promoting the state of inflammation [41, 42].

Interestingly, genetic factors are associated with monozygotic Finnish twins [43]. Several genetic polymorphisms are associated with an increased risk, including PNPLA3 (I148M) and PNPLA3 (S453I), which function the triacylglycerol lipase [44] and acylglycerol transacylase and NCAN, a cell adhering molecule [45]. These genetic variations might explain the potential reasons why some people tend to be obese [37].

## 3 Research Design and Methods

### 3.1 Registration of Review Protocol

The protocol for this systematic review was registered and available in the Open Science Framework (10.17605/OSF.IO/2V6RD).

### 3.2 Data Sources and Searches

Studies were included if they were original studies that reported the incidence rates of weight change in adult individuals (> 18 years old) with NAFLD compared to those without NAFLD. The study participants were of either sex with no restrictions in terms of ethnicity and comorbidities. Based on data from eligible studies, “severe” NAFLD was defined either by the presence of increasing ultrasonographic steatosis scores or by a high NAFLD fibrosis score (NFS), which is a reliable noninvasive marker of advanced NAFLD fibrosis [1].

**Figure 1:**
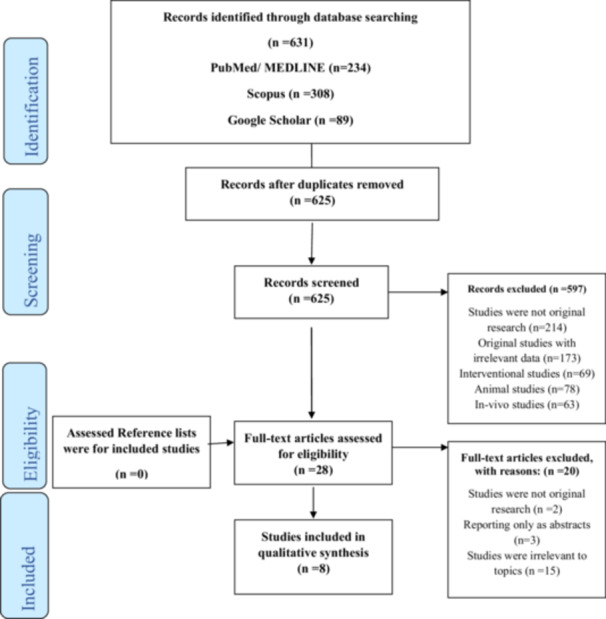
Progress through stages of systematic review [46].

No language restrictions were applied. The exclusion criteria of the systematic review were as follows: (1) reviews, editorials, abstracts, case reports, practice guidelines, and randomized controlled trial studies; (2) studies that used only serum liver enzyme levels, fatty liver index, or other surrogate markers to diagnose NAFLD; (3) studies conducted in the pediatric or specific populations (< 18 years old); (4) studies showing change in weight by surgery; and (5) studies that did not report any hazard ratio (HR) and 95% confidence interval (CI) for the outcome of interest (incident change in weight). The reference lists of relevant and previous review articles were manually searched for other relevant studies. The included and excluded studies were collected following the Preferred Reporting Items for Systematic Reviews (PRISMA) flow diagram (Figure 1).

The search strategiy is listed in Table 1. A complete search of the references of the retrieved articles was performed by one of the authors (N. D) to identify all the related articles.

**Table 1:** Search strategies for PubMed and Scopus data bases.

Search engine	Search strategy	Additional filters
PubMed/MEDLINE	(“Body Weight”[tiab] OR “Body Mass Index”[tiab] OR “Body Weight”[Mesh] OR “Body Mass Index”[Mesh]) AND (“fluctuation”[tiab] OR “change”[tiab] OR “variability”[tiab] OR “increase”[tiab] OR “decrease”[tiab] OR “loss”[tiab] OR “gain”[tiab] OR “cycling”[tiab]) AND (“Non‐alcoholic Fatty Liver Disease”[tiab] OR “Non‐alcoholic Fatty Liver Disease”[Mesh]) AND (“Risk”[tiab] OR “Probability”[tiab] OR “Risk”[Mesh] OR “Probability”[Mesh]) AND (“Population”[tiab] OR “Population Groups”[tiab] OR “General Population”[tiab] OR “Population”[Mesh] OR “Population Groups”[Mesh] OR “General Population”[Mesh])	English
Scopus	((TITLE‐ABS‐KEY (body AND weight) OR TITLE‐ABS‐KEY (body AND weights) OR TITLE‐ABS‐KEY (weight, AND body) OR TITLE‐ABS‐KEY (weights, AND body))) AND ((TITLE‐ABS‐KEY (fluctuation) OR TITLE‐ABS‐KEY (change) OR TITLE‐ABS‐KEY (variability) OR TITLE‐ABS‐KEY (increase) OR TITLE‐ABS‐KEY (decrease) OR TITLE‐ABS‐KEY (loss) OR TITLE‐ABS‐KEY (gain) OR TITLE‐ABS‐KEY (cycling))) AND ((TITLE‐ABS‐KEY (Non‐alcoholic AND fatty AND liver AND disease) OR TITLE‐ABS‐KEY (non AND alcoholic AND fatty AND liver AND disease) OR TITLE‐ABS‐KEY (nafld) OR TITLE‐ABS‐KEY (nonalcoholic AND fatty AND liver AND disease) OR TITLE‐ABS‐KEY (fatty AND liver, AND nonalcoholic) OR TITLE‐ABS‐KEY (fatty AND livers, AND nonalcoholic) OR TITLE‐ABS‐KEY (liver, AND nonalcoholic AND fatty) OR TITLE‐ABS‐KEY (livers, AND nonalcoholic AND fatty) OR TITLE‐ABS‐KEY (nonalcoholic AND fatty AND liver) OR TITLE‐ABS‐KEY (nonalcoholic AND fatty AND livers) OR TITLE‐ABS‐KEY (nonalcoholic AND steatohepatitis) OR TITLE‐ABS‐KEY (nonalcoholic AND steatohepatitides) OR TITLE‐ABS‐KEY (steatohepatitides, AND nonalcoholic) OR TITLE‐ABS‐KEY (steatohepatitis, AND nonalcoholic))) AND ((TITLE‐ABS‐KEY (risk) OR TITLE‐ABS‐KEY (risks) OR TITLE‐ABS‐KEY (relative AND risk) OR TITLE‐ABS‐KEY (relative AND risks) OR TITLE‐ABS‐KEY (risk, AND relative) OR TITLE‐ABS‐KEY (risks, AND relative) OR TITLE‐ABS‐KEY (probability) OR TITLE‐ABS‐KEY (probabilities))) AND ((TITLE‐ABS‐KEY (population) OR TITLE‐ABS‐KEY (populations) OR TITLE‐ABS‐KEY (general AND population))) AND (LIMIT‐TO (LANGUAGE, “English”))	English

EndNote 8X software was used to manage the retrieved references. Duplicate articles were excluded. The screening was initially conducted by titles (D.A and M.N) and then abstracted by two authors separately (N.R; N.N). In the next step, the full text of the articles was evaluated by two independent reviewers (M.A.A; N.D.). Working independently and in duplicates, we read the articles and determined whether they met the inclusion criteria. Discrepancies were resolved by consensus and then by referring to the original article in consultation with a third author (N.D.).

### 3.3 Data Extraction and Quality Assessment

For all studies, we extracted information on the study characteristics, type of study, follow‐up duration, population characteristics, type of exposure, exposure definition, assessment methods, outcome of interest, adjusted variables, and quality assessment score. Any disagreements or disputes between the two evaluators were settled by agreement and, then a third evaluator (N.D.), and lastly. Two authors (D.A. and M.N.) independently assessed the risk of bias. The JBI Critical appraisal tools for cohort and cross‐sectional studies (jbi. global/critical‐appraisal‐tools) were used to judge the quality of each type of study. We judged studies that received a score of one‐half of the total score to be at low risk of bias, as provided in the supplementary file, and those that scored less than one‐half of the total score to be at high risk and eliminated.

### 3.4 Statistical Analysis

All analyses were performed using STATA version 17 and R version 4.4. Proportional meta‐analysis was used in this study. To evaluate the association between NAFLD and weight change, we used the pooled odds ratio (OR) and a 95% CI to determine the strength of their association. This approach allowed us to quantify the relationship between NAFLD and weight change and provided a measure of statistical certainty.

To validate our results, we checked for confounding factors that may have affected our analyses using a meta‐regression. We performed a leave‐one‐out sensitivity analysis to evaluate the effects of studies with extreme results. To explore statistical significance, results with a two‐tailed *p*‐value of less than 0.05 were considered significant. In exploring the heterogeneity of the studies included in the analysis, *I* ^2^ and Cochrane Q statistics were used, with an *I* ^2^ value greater than 50% indicating substantial heterogeneity and a *p*‐value less than 0.10 on the Q test indicating statistical significance. Given the heterogeneity of the data, we employed a random‐effects model for all the analyses.

### 3.5 Finding and Results

In this study, 631 records were obtained from the databases. After removing six duplicated records, we screened 625 citations and assessed their titles and abstracts. After reviewing the titles and abstracts and excluding irrelevant articles, 28 articles remained for the full‐text assessment, and a further 20 were excluded with reasons. Finally, eight studies [47, 48, 49, 50, 51, 52, 53, 54] met our inclusion criteria, and no articles were found by reviewing the reference list of the included studies. Only studies with fair to good quality were included.

The findings and main characteristics of the included studies are presented in Table 2. These articles assessed the effect of weight change on NAFLD incidence in 147,601 participants and were published between 2005 and 2022. Three studies were conducted in the United States [48, 49, 52], two in Korea [47, 54] and Japan [50, 51], and one in China [53]. We only included cohort and cross‐sectional studies. Five studies used cohort design [47, 48, 49, 53, 54], and three were cross‐sectional [50, 51, 52]. Of the eight studies, two studies used participants' self‐reported data [49, 51], five used measuring weights [47, 48, 52, 53, 54], and one measured the present weight but used self‐reported weight at age 20 to assess weight changes [50]. Most studies have used body weight change in kg to define the weight change [47, 49, 50, 51, 52]. Two studies used BMI [48, 53], and only one study used body weight change percentage [54]. All cohort studies had a quality score of 8 or above, with two studies getting a total score of 11/11, and all cross‐sectional studies scored at least 6 out of 8.

**Table 2:** Description of studies included in the study.

First author (year) (reference)	Type of study	Country	Follow‐up duration	Participants	Sex	Mean age (year)	Type of exposure	Exposure definition	Assessment method measured/self‐report	Adjusted variables	Outcome	Quality score
Mi Na Kim (2020) [49]	Cohort	USA	20 years	Female, registered nurses	*N* Total = 110,054	40.91	Weight change	Weight loss ≥ 2.0 kg; Loss or gain within 2.0 kg; Gain 2.0–5.9 kg; Gain 6.0–9.9 kg; Gain 10.0–19.9 kg	Self‐reported	age, BMI at age 18 years, smoking status (never, past, current), total calories (kcal), menopausal hormone therapy (never, past, current, premenopausal), Alternate Healthy Eating Index score (quintiles, missing), physical activity (quintiles), type 2 diabetes (yes or no), hypertension (yes/no), dyslipidemia (yes or no), physical activity (MET‐hours/week, quintiles), regular use of aspirin (yes/no), race (White/non‐White)	Women with ≥ 20 kg of adulthood weight gain had the multivariable aHR of 6.96 (95% CI, 5.27–9.18), and this remained significant after further adjusting for early adultood BMI and updated BMI (*p* trend < 0.0001)	
					Female (110,054, (100%)						(Weight gain ≥ 20 kg aHR: 6.96 (95% CI, 5.27–9.18) Weight gain 10–19.9 kg aHR: 2.99 (95% CI, 2.26–3.97) Weight gain 6.0–9.9 kg aHR: 1.59 (95% CI, 1.17–2.16) Weight gain 2.0–5.9 kg aHR: 1.26 (95% CI, 0.91–1.74) Weight gain 2.0 kg: 1 (reference)	11/11
								Gain ≥ 20.0 kg			Weight loss ≥ 2.0 kg aHR: 1.26 (95% CI, 0.88–1.82))	
											The age‐adjusted incidence rate of NAFLD	
Yuqing Ding (2022) [48]	Retrospective cohort	USA	10 years	Civilian United States population	*N* Total = 2212	55.74 ± 0.47	Weight change	Stable nonobese (BMI age 25 < 30 kg/m^2^, BMI 10 prior < 30 kg/m^2^ and BMI baseline < 30 kg/m^2^), Early adulthood weight gain (BMI age 25 < 30 kg/m^2^, BMI 10 prior ≥ 30 kg/m^2^ and BMI baseline ≥ 30 kg/m^2^), Middle and late adulthood weight gain (BMI age 25 < 30 kg/m^2^, BMI 10 prior < 30 kg/m^2^ and BMI baseline ≥ 30 kg/m^2^), Revert to nonobese (BMI age 25 < 30 kg/m^2^, BMI 10 prior ≥ 30 kg/m^2^ and BMI baseline < 30 kg/m^2^)	Measured	Age, sex, race/ethnicity, education level, family income–poverty ratio level, marital status, alcohol consumption and smoking status, chronic diseases, leisure time physical activity level, HEI	67.20% (95% CI 58.75–75.64%) in the “early adulthood weight gain” group 54.57% (95% CI 46.82–62.31%) in the “middle and late adulthood weight gain” group 37.40% (95% CI 27.85–46.95%) in the “revert to the nonobese” group 23.82% (95% CI 19.66–27.98%) in the “stable nonobese” group. Compared with the	11/11
					Female (1156; 53.78%)						Stable nonobese participants, those who gained weight in early adulthood were associated with a 119% higher risk of NAFLD (RR 2.19, 95% CI 1.64–2.91) and those who gained weight	
											At middle and late adulthood had 92% higher risk of NAFLD (RR 1.92, 95% CI 1.40–2.62). Notably, the “revert to nonobese” group showed a null association with NAFLD, with RR of 1.01 (95% CI 0.62–1.64)	
								Two main groups:			Weight gain increased the incidence of NAFLD for 1.22 (1.16–1.29)	
Jing wu, (2018) [53]	Cohort	China	6 years	Chinese population	*N* Total = 646	46±	Gain weigh	1. BMI > 24.7 2. BMI < 24.7	Measured	Age and gender	The association between changing in body weigh and FLD: (odds ratio (OR) = 1.56; 95% confidence interval (95%CI): 1.27–1.92	9/11
					Female (375; 58%)							
Se ho park (2005) [54]	Cohort	Korea	1 year	Korean men	*N* Total = 615	41.11±	Weigh change	Body weigh change	Measured	BMI, CRP, FBS, F.Insulin	NAFLD, had a significantly higher prevalence in the AWG group (matched AWG group vs. matched non‐AWG group; 47(9.8%) vs. 79(16.4%), *p* =) 0.003).	8/11
					Male (615; 100%)			> 5% < 5%		GGT, HDL, HOMA‐IR, LDL, TC, TG, WC		
Sho Tanaka (2020) [38]	Cross‐sectional study	Japan	1 year	General population	*N* Total = 3503	49.5 ± 11.3	Weight change	AWG ≥ 10 kg AWG ≤ 10 kg	Self‐reported	ALT, AST, BMI, BP, eGFR, G‐GTP, HbA1c, HDL‐C, LDL‐C, TG, WC	The association between weight gain and NAFLD remained significant after adjustment for demographic and metabolic risk factors (OR 1.87, 95%CI 1.19–2.95 for CAP score ≥ 263 dB/m; OR 2.23, 95%CI 1.48–3.35 for CAP ≥ 285 dB/m)he association between weight gain and NAFLD remained significant after adjustment for demographic and metabolic risk factors (OR 1.87, 95%CI 1.19–2.95 for CAP score ≥ 263 dB/m; OR 2.23, 95%CI 1.48–3.35 for CAP ≥ 285 dB/m)t	8/8
					Female (1916; 59.62%)							
Karn Wijarnpreecha (2022) [52]	Cross‐sectional study	USA	1 year, 10 years	General population (adults over 36 years)	*N* Total = 4829	56.575 + 0.675	Weight change	Quartile 1, <−7 (−3.18 kg) pounds Quartile 2, −7 pounds (−3.18 kg) to 6 (2.72 kg) pounds Quartile 3, 6 pounds (2.72 kg) to 20 pounds Quartile 4, ≥ 20 pounds (9.07 kg)	Measured	Age, sex, race/ethnicity, education level, marital status, smoking status, hypertension, diabetes, TC, HDL‐C, and leisure‐time physical activity, WC.	In terms of significant fibrosis, multivariate‐adjusted OR for significant fibrosis were 1.99 (95%CI 1.05–3.79 for the third quartile) and 3.12 (95%CI 1.46–6.65 for the fourth quartile), respectively. A statistically significant association between weight gain over 1 year and NAFLD was noted, whereas no such association was found between weight gain and significant fibrosis.	8/8
					Female (53.575%)							
Yoo Soo Chang (2009) [55]	Cohort	Korea	5 years	Korean men workers	*N* Total = 4246	36.7 ± 4.8	Weight change	<−0.9 kg −0.9 to 0.5 kg 0.6–2.2 kg ≥ 2.3 kg	Measured	Age, smoking status, the period from visit 1 to visit 2, BMI, HDL‐C, triglycerides, ALT, uric acid, HOMA‐IR	aHR for:	8/11
					Men = (4246; 100%)						Q1: 0.9 (0.71–1.15) Q2: 1.00 (reference) Q3: 1.10 (0.88–1.36) Q4: 1.26 (1.01–1.58)	
Takeshi Kimura (2015) [50]	Cross‐sectional	Japan	—	General population	*N* Total = 21496	47.4 ± 11	Weight change	Men:	Measured	Age, BMI at age 20, alcohol consumption, smoking status, exercise, FBG, TG, uric acid, ALT	Men	8/8
											OR for:	
											BW loss: 0.26 (0.2–0.35) Q1: 1 (reference) Q2: 2.34 (1.94–2.83) Q3: 5.36 (4.46–6.43) Q4: 18.24 (15.10–22.03)	
								−0.1 kg ≥ (BW loss group) 0–4.1 kg 4.1–7.7 kg 7.7–12.1 kg ≥ 12.1 kg			Women:	
								Women			OR for:	
					Female (13,486; 62.8%)			−0.1 kg (BW loss group)≥ 0–2.1 kg 2.1–4.5 kg 4.5–8.2 kg ≥ 8.2 kg			BW loss: 0.20 (0.13–0.3) Q1: 1 (reference) Q2: 1.59 (1.13–2.26) Q3: 4.02 (2.95–5.47) Q4: 18.69 (14.0–24.93)	

Abbreviations: ALT, alanine aminotransferase; AST, aspartate aminotransferase; AWG, adult weight gain; BMI, body mass index; BP, blood pressure; CRP, C‐reactive protein; F‐insulin, fasting plasma insulin; FBS, fasting blood sugar; eGFR, estimated glomerular filtration rate; GGT, γ‐glutamyl transferase; G‐GTP, gamma‐glutamyl transpeptidase; HbA1c, glycated hemoglobin; HDL, high density lipoprotein cholesterol; HDL‐C, high‐density lipoprotein cholesterol; HEI, healthy eating index scores; HOMA‐IR, homeostasis model assessment of insulin resistance; LDL, low density lipoprotein cholesterol; LDL‐C, low‐density lipoprotein cholesterol; MET, metabolic equivalent of tasks; NAFLD, nonalcoholic fatty liver disease; TC, total cholesterol; TG, triglycerides; WC, waist circumference.

All the participants were adults. The participants of five studies were among the general population [48, 50, 51, 52, 53]. Both Korean studies were conducted on Korean men [47, 54], and one study was on women [49].

Most studies reported that weight gain per se was a risk factor for NAFLD incidence, which contributed to the increase in the risk of developing NAFLD even after adjusting multiple covariates [47, 48, 49, 50, 51, 52, 53]. Of note, weight gain during early adulthood and the 20 s significantly contributed [48, 49, 50]. Subjects who gained weight during middle or late adulthood had a higher risk of developing NAFLD than stable nonobese participants but less risk than those who gained weight during early adulthood, which shows that NAFLD was also associated with obesity or weight gain in middle or late adulthood, but the contribution of weight change in the youth was more prominent [48].

The trajectory of body shape and weight during a lifetime of 10 years is a risk factor for NAFLD [49, 52]; therefore, the emphasis was on maintaining a stable and lean weight throughout life [48, 49]. Interestingly, according to both Japanese studies, the lean population with normal BMI was more susceptible to developing NAFLD if they gained weight [4, 6], highlighting the importance of monitoring body weight in normal and healthy populations. Furthermore, it was found that reducing body weight was a critical factor in fatty liver disease improvement [54], and men with mild weights at the baseline had a higher chance of NAFLD remission [53].

### 3.6 Weight Change and the Risk of NAFLD

**Figure 2:**
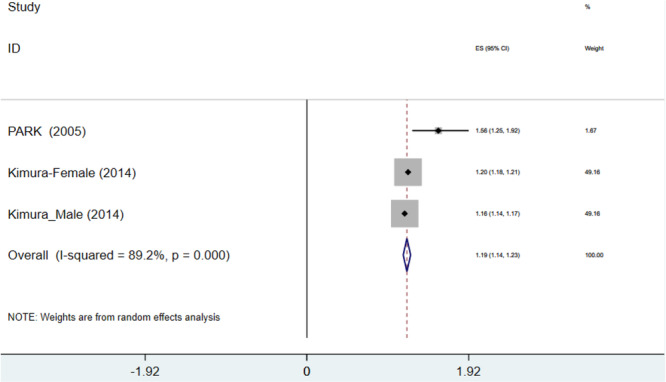
Forest plot of association between weight decrease and nonalcoholic fatty liver disease (NAFLD) odds ratio (OR). CI, confidence interval.

**Figure 3:**
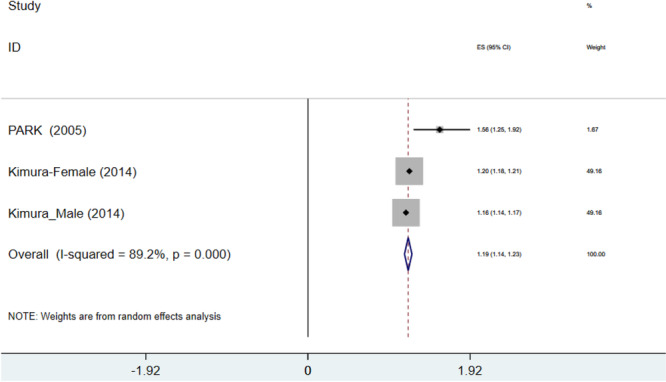
Forest plot of association between weight decrease per 1 kg and nonalcoholic fatty liver disease (NAFLD) odds ratio (OR). CI, confidence interval.

Reporting the relationship between weight loss and risk of NAFLD in four studies (one study reported a separation between males and females) showed a pooled OR of 1.16 (OR = 1.16, 95% CI: 1.118–1.208, *p* < 0.0001, *I* ^2^ = 86%) (Figure 2). Therefore, our analysis revealed that patients experiencing weight loss had a 1.16‐fold increased risk of NAFLD compared to controls (subjects with stable weight during their adulthood). Conversely, three studies showed that every 1 kg of weight loss was associated with a 1.186‐fold increased likelihood of NAFLD when compared to the control group (OR = 1.186, 95% CI: 1.142–1.230, *p* < 0.0001, *I* ^2^ = 89.2%) (Figure 3).

**Figure 4:**
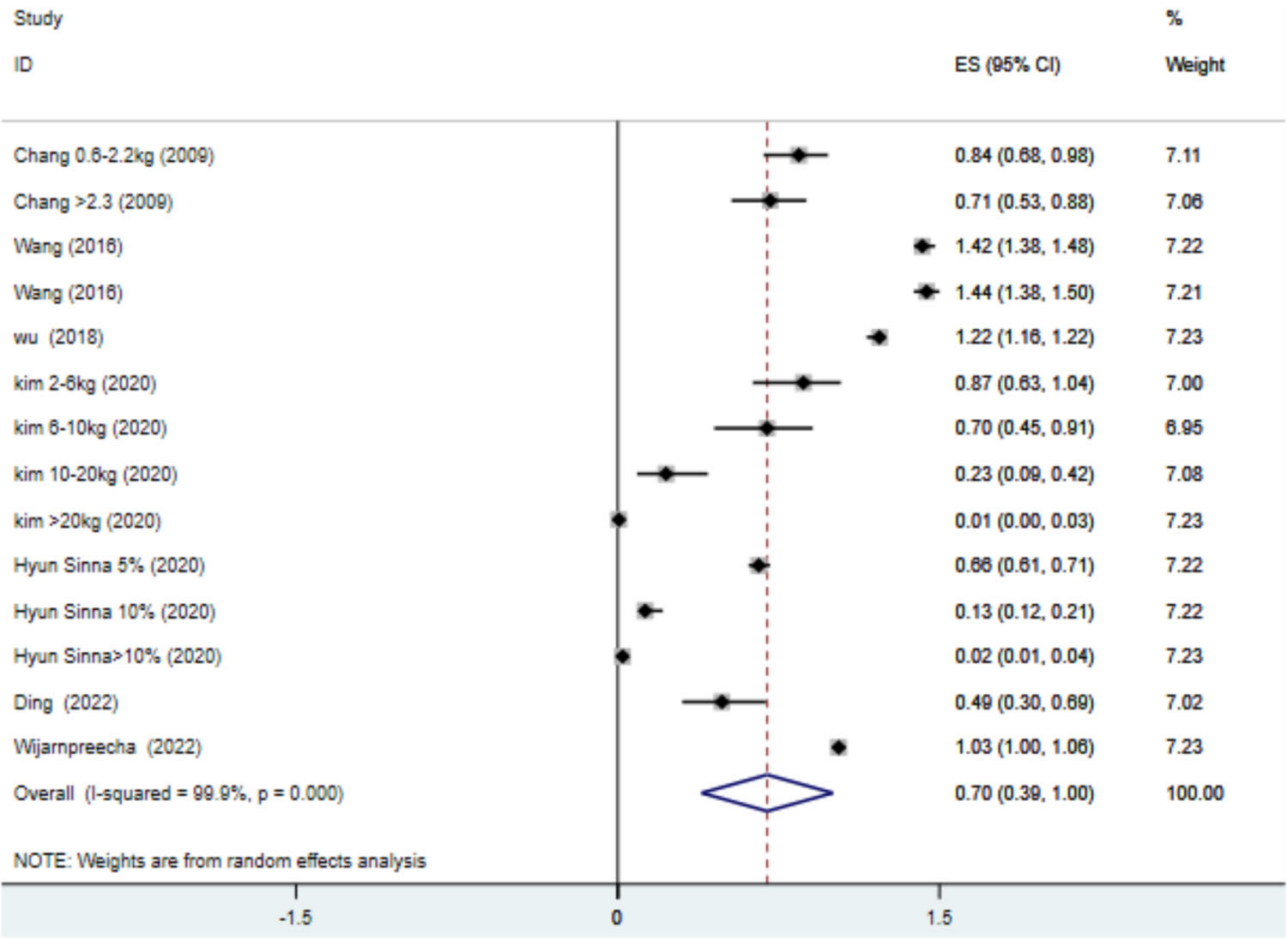
Forest plot of association between weight increase and nonalcoholic fatty liver disease (NAFLD) odds ratio (OR). CI, confidence interval.

**Figure 5:**
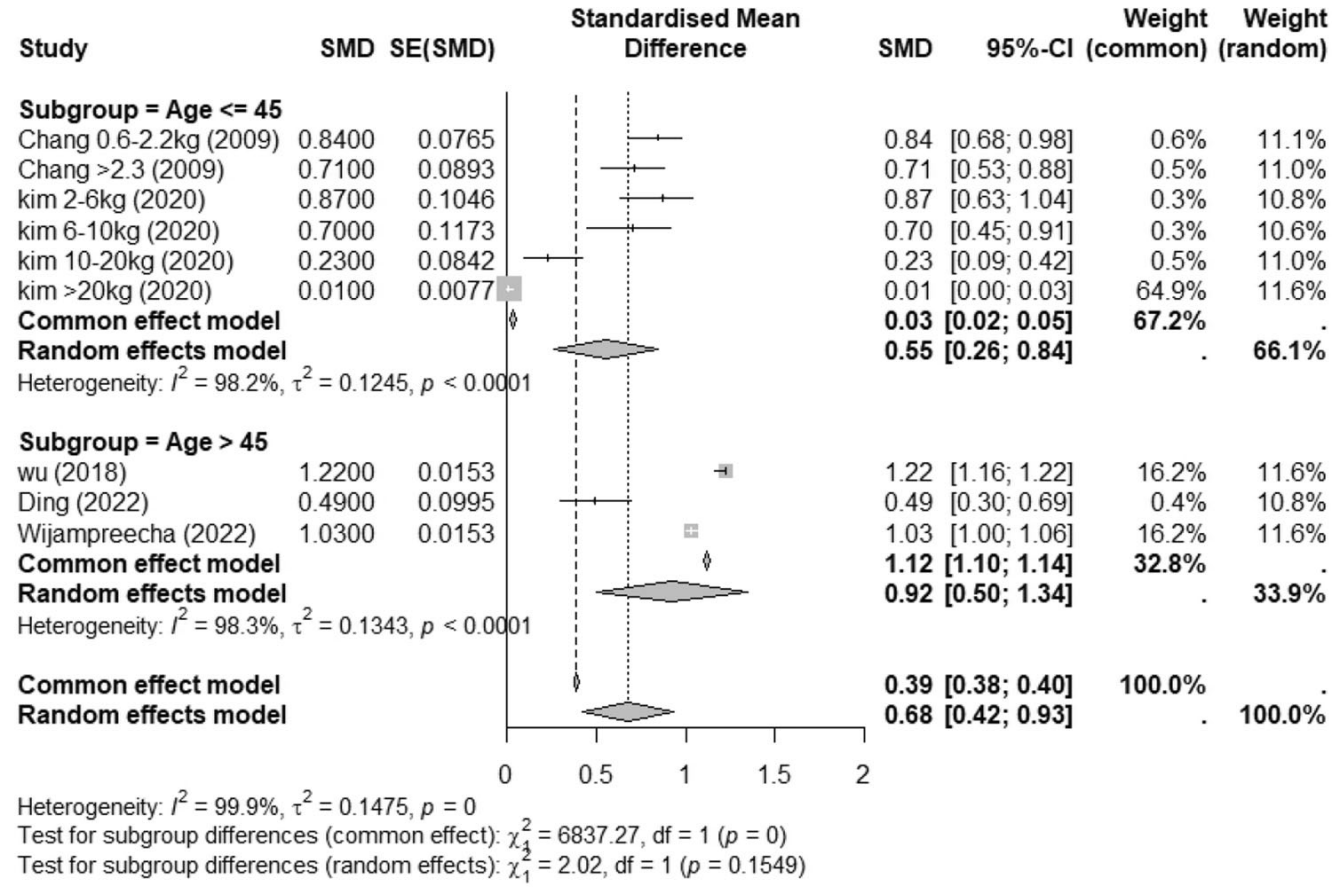
Forest plot (mean age subgroup) of association between weight increase and nonalcoholic fatty liver disease (NAFLD) odds ratio (OR). CI, confidence interval.

The findings of seven articles indicated that the odds of experiencing NAFLD in patients with increased weight in a wide range (< 1 kg to > 20 kg) was 0.697 (OR = 0.697, 95% CI: 0.391–1.002, *p* < 0.0001, *I* ^2^ = 99.9%) (Figure 4), and the findings of three articles indicated that the odds of incident NAFLD in patients with increased weight in a wide range (< 1 kg to > 20 kg who were aged > 45 years was 0.92 (OR = 0. 92, 95% CI: 0.50–1.34, *p* < 0.0001, *I* ^2^ = 98.3%) (Figure 5) suggesting a slightly reduced likelihood compared to the control group. Our study was limited by the relatively high heterogeneity observed among the included studies, necessitating a random effects analysis. Variations in demographic characteristics and weight change reporting methodologies may result in heterogeneity. The overall heterogeneity, as measured by the *I* ^2^ statistic, remained essentially unchanged across the sensitivity analysis, ranging from 86% to 99.9% (Supporting Information: Figure 1).

## 4 Discussion

The relationship between weight change and NAFLD has been a subject of considerable interest in the scientific literature, given the significant impact that the coexistence of any weight fluctuations can have on the overall prognosis of individuals. The growing body of research in this area underscores the importance of understanding and investigating the association between weight fluctuations and NAFLD to enhance our knowledge of their interconnectedness in patients.

This systematic review of eight studies summarized the evidence for the association between the change and fluctuation in weight and the risk of NAFLD. We showed that weight change had a significant effect on the risk of NAFLD. Interestingly, weight gain was not a risk factor for NAFLD in this study. Nevertheless, maintaining a stable and lean weight throughout life and experiencing minimum weight change has been a suggested way of preventing NAFLD. According to a systematic review and meta‐analysis of 22 randomized clinical trials that included 2588 patients with NAFLD, weight loss could improve biomarkers of liver disease [56]. Considering the positive effects of weight loss on NAFLD, our study also suggests that weight loss might not be helpful per se; however, minimum weight fluctuation may be considered a better prognostic variable. It is essential to note that this observation has not been consistently supported by other studies, leading to conflicting results.

A cohort of 615 men with NAFLD and elevated alanine aminotransferase Park et al. [54] reported that extensive changes in anthropometric indices and metabolic parameters were similarly associated with fatty liver disease. The authors proposed that weight loss is the key to improving fatty liver disease. Several studies have consistently emphasized the role of weight gain. Tanaka et al. [51] suggest that anthropometric parameters had significant associations with NAFLD, and adult weight gain was a significant risk factor for developing NAFLD. Interestingly, Wu et al. [53] concluded that the baseline BMI measurements were, per se, associated with his NAFLD remission. Wijarnpreecha et al. [52] concluded that adults who gained weight over ten years had higher odds of developing NAFLD and liver fibrosis. This result supports the hypothesis of the simultaneous effect of high speed, a large amount of weight loss, and continuous weight gain over time in the NAFLD incidence. In another study, Ding et al. [48] reported that weight gain throughout adulthood in nonobese people was independently linked to a higher chance of developing NAFLD, and losing weight later in life will likely reduce the risk of NAFLD significantly. When taken as a whole, this study offered quantitative estimates showing that individual weight‐maintenance techniques may help slow the course of NAFLD and its related complications. Chang et al. [47] concluded that weight gain is significantly associated with USFL development. In their study, weight gain, even in normal‐weight participants, was an independent predictor of USFL in a dose‐dependent manner. The relationship between weight gain and increased USFL risk was independent of the baseline BMI and various confounding factors.

Moreover, weight gain consistently predicted future USFL even after considering updated values of BMI and other covariates at each follow‐up. These results suggest that, in addition to BMI, weight gain itself is a risk factor for USFL and may have adverse health effects. According to a cross‐sectional study by Kimura et al. [50], the prevalence of NAFLD increased in proportion to weight gain after 20 years of age. The effects of adult weight gain are powerful in people who are currently of normal weight, a group of patients traditionally thought to be at a low risk for lifestyle‐related diseases. Longitudinally monitoring body weight in apparently healthy individuals for the primary prevention of NAFLD and other lifestyle‐related diseases. The large prospective cohort of US women by Kim et al. [49] concluded that excessive obesity and weight gain from early adulthood were associated with a significantly higher risk of developing NAFLD in middle age.

A single‐center retrospective study was done by V. DiBattista et al. [57] on 11,559 patients with NAFLD. In 1–2 years, more than half of the NAFLD patients experienced 5% weight loss or gain, and these changes were usually sustained for up to 4–5 years. This result confirmed that the weight change can independently increase the risk of NAFLD. Rapid weight loss has been reported to induce hepatic inflammation and exacerbate steatohepatitis [58, 59].

In another study by Jung et al. [60] on health checkup data of 30,708 participants, those in the highest quartile of average successive variability of weight (ASVW) showed a significantly increased risk of NAFLD compared to those in the lowest quartile. Participants with increased weight over 4 years and high ASVW demonstrated a higher risk of NAFLD than those with stable weight and low ASVW.

The mechanism by which weight gain increases the risk of NAFLD and, consequently, liver fibrosis, is not completely clear yet. However, as mentioned earlier, several lines of evidence show that insulin resistance, lipotoxicity, inflammation, and oxidative stress are linked to disease progression [61, 62, 63]. Dysregulation of adipokines, such as leptin, a pro‐fibrogenic cytokine, is also responsible for the mechanism of NAFLD caused by obesity [64].

Excessive free fatty acids (FFAs) have been shown to activate various immune cells and, therefore, cause hepatocyte apoptosis [65]. Moreover, cell debris, pro‐inflammatory adipokines, and cytokines can increase the secretion of tumor necrosis factor (TNF) and IL‐6 from Kupffer cells, leading to the activation of downstream signaling molecules, such as STAT3 in hepatocytes, which contribute to the inflammatory state and carcinogenesis [66].

Triglyceride accumulation in hepatocytes leads to hepatosteatosis. The resolution or improvement of the chronic inflammatory state related to obesity can result from achieving and then maintaining appropriate weight control. The poor insulin resistance and complications associated with obesity, as mentioned earlier, can be resolved by weight reduction in the obese population [67]. This can be explained by the inflammatory state of obesity and the role of adipokines in inflammation [68]. Interestingly, weight loss can improve dysbiosis and maintain gut microbiota, which can be beneficial for lowering the inflammatory state [69, 70].

According to Tarantino et al., patients with BCa consistently had NAFLD. The TyG Index was used to assess the existence of NAFLD, which was evident in both groups; however, the BCa group had a significantly higher prevalence of NAFLD than the no‐Ca BD group. Similarly, the BCa group had higher rates of prediabetes and T2DM than the no Ca BD group. In both groups, age was a reliable predictor of glucose metabolism abnormalities, and in the BCa group, it also affected grading and staging. Triglyceride/HDL ratio and TyG index also supported the finding that IR was more common in the BCa group than in the no Ca BD group. It is interesting to note that the median TyG Index value of BCa patients was 4.66, above the cut‐off of 4.59 mg/dL (the index of the existence of NAFLD), indicating that this restriction was a suitable. TyG index showed good reliability and was a reliable predictor of glucose homeostasis.

Concerning the performance of the AST/ALT ratio, it is essential to emphasize that liver enzymes are both vague and insensitive indicators of NAFLD; as a result, these tests cannot demonstrate the existence of NAFLD. In fact, most (up to 80%) subjects with NAFLD have average serum liver enzyme concentrations, even though NAFLD is the most common cause of elevated serum ALT and/or AST levels [5]. This is a critical comorbidity that warrants further consideration in prospective studies. The higher prevalence of NAFLD, IR, prediabetes, and T2DM in the BCa group indicates the need for these disorders to be considered as adjunct factors that could impact this cancerous disease [5, 71].

Obesity is associated with inflammation, which is a response to noxious stimulants. Chronic low‐grade inflammation is considered a trigger for metabolic syndrome and its complications. Weight loss can reduce the serum inflammatory indices and improve insulin sensitivity. Chronic inflammation in obesity and metabolic syndrome is a low‐grade inflammatory condition, meta‐inflammation, or para‐inflammation. Studies have shown a positive correlation between MPV, BMI, and the activation of the inflammatory system in type 2 diabetes mellitus.

Inflammatory markers may also reflect the level of control of type 2 diabetes. A study found a strong correlation between MPV and HbA1c levels in well‐controlled diabetics, suggesting strength MPV to state inflammation in type 2 diabetes mellitus. However, the study has limitations, including the small cohort and retrospective design. Obesity is linked to inflammation, which is a response to noxious stimulants. Chronic low‐grade inflammation is considered a trigger of the metabolic syndrome and its complications. Weight loss can reduce serum inflammatory indices and improve insulin sensitivity. Chronic inflammation in obesity and metabolic syndrome is called a low‐grade inflammatory condition, meta‐inflammation, or para‐inflammation. Studies have shown a positive correlation between MPV, BMI, and activation of the inflammatory system in type 2 diabetes mellitus [72, 73].

Inflammatory markers may also reflect the control level of type 2 diabetes. A study found a strong correlation between MPV and HbA1c level in well‐controlled diabetics, suggesting MPV′s strength to state inflammation in type 2 diabetes mellitus. However, the study has limitations, including a small cohort and retrospective design. The main finding of the retrospective analysis study by Aktas et al. is that MPV could be a marker of the inflammatory burden of glycemic control and obesity in patients with type 2 diabetes mellitus [74].

This systematic review was a comprehensive study. According to the articles reviewed in this study, there was a significant association between the risk of NAFD and the effects of weight change, or variation. One of the strengths of this study was the strong search strategy and diversity of the databases to ensure that any potentially relevant records were included.

This study has some limitations. First, a particular risk of selection bias remained, even though the main parameters of the replicate population did not differ from those of the entire population. Second, the presence or absence of NAFLD was diagnosed by ultrasound, which may be less sensitive than liver biopsy. In addition, ultrasonography cannot detect inflammation or fibrosis. Third, information on exercise and alcohol consumption was obtained only by directly interviewing participants. Fourth, histological evidence (i.e., biopsy) of NAFLD was not included because it is not appropriate to perform invasive testing in population‐based epidemiological studies. However, fatty deposits in the liver can be diagnosed noninvasively using US or other imaging modalities, and many population‐based studies have used ultrasound. Fifth, alcohol consumption was self‐reported and may be have been underestimated. Sixth, this study acknowledges the controversy surrounding the association between obesity and NAFLD. While some existing high‐level evidence, including randomized controlled trials and longitudinal cohort studies, supports this association, the studies included in this systematic review—comprising primarily cross‐sectional studies and cohort studies—reflect lower levels of evidence. This represents a limitation of our study, which we aimed to address by emphasizing that our findings do not negate the well‐established role of weight gain in increasing NAFLD risk. Instead, we suggest that weight variability may play an additional pathophysiological role in this complex association. Further studies with higher levels of evidence are required to substantiate this hypothesis and provide more definitive conclusions.

## 5 Conclusion

This systematic review examined potential associations between the risk of NAFD and the effects of weight change or variation. This study revealed an increased risk of developing NAFD caused by weight loss. Conversely, weight gain had no specific effect on the development of NAFLD. Future prospective studies are necessary to assess the relationship between the impact of speed and rate of change, fluctuation, or change in weight and NAFD. The current results indicate that high weight levels should be controlled and abnormal weight fluctuations must be prevented to restore regular weight rhythm. However, the precise mechanism through which weight gain is associated with NAFLD development remains unclear. Therefore, we suggest future preclinical studies to assess this pathology. We suggest clinical research focusing on treatment approaches for weight reduction specific to high‐risk individuals who gain weight. Finally, additional large‐scale studies in racially and ethnically diverse populations of men and women are required to further assess the association between weight fluctuations and NAFLD development. To mitigate the risk of NAFLD development and improve overall cardiovascular health, strong emphasis should be placed on health promotion activities such as weight loss programs. In addition, to ensure easy and equitable access for all, it is also vital that public health professionals pay attention to the complicated role of social and ecological factors on the effectiveness of care promotion activities between racial groups.

## Author Contributions

**Dorsa Alijanzadeh:** writing – original draft, writing – review and editing. **Masoud Noroozi:** conceptualization. **Negin Rostami:** data curation, formal analysis, writing – review and editing. **Narges Norouzkhani:** conceptualization, data curation, investigation, methodology, writing – original draft, writing–review and editing. **Mahdie ShojaeiBaghini:** data curation, writing – review and editing. **Saeed Zivari Lashkajani:** methodology, writing – review and editing. **Ali Mirzaei:** conceptualization, writing – review and editing. **Maryam Dianati:** writing – review and editing. **Maryam Salimi:** writing – review and editing. **Hamidreza Sadeghsalehi:** formal analysis, writing – review and editing. **Faezeh Jadidian:** data curation, writing – review and editing. **Sajjad Ghane Ezabadi:** conceptualization, writing – original draft. **Mohammad Sadegh Fallahi:** writing – review and editing. **Mobina Fathi:** writing – review and editing. **Alaleh Alizadeh:** writing – review and editing. **Mahsa Asadi Anar:** project administration, writing – original draft. **Niloofar Deravi:** project administration, writing – original draft.

## Ethics Statement

The authors have nothing to report.

## Conflicts of Interest

The authors declare no conflicts of interest.

## Transparency Statement

The lead author Mobina Fathi, Alaleh Alizadeh, Mahsa Asadi Anar, Niloofar Deravi affirms that this manuscript is an honest, accurate, and transparent account of the study being reported; that no important aspects of the study have been omitted; and that any discrepancies from the study as planned (and, if relevant, registered) have been explained.

## Supporting information


**Supplementary Figure 1:** Meta‐analysis random‐effects estimates of association between weight increase and NAFLD OR.

## Data Availability

Data is available upon request from corresponding author. All data generated or analyzed during this study are available at each database through the same searching strategy included in this published article and its supplementary information files. The variables and relationships examined in the present article have not been examined in any previous or current articles, or to the best of our knowledge in any papers that will be under review soon.
