# Supplementary material for: Impact of Change, Fluctuation, or Variability in Weight on the Risk of Nonalcoholic Fatty Liver Disease in General Population: A Systematic Review and Meta‐Analysis
Source: Health Sci Rep. 2025 Sep 29;8(10):e71255. doi: 10.1002/hsr2.71255 (PMC12477495; doi:10.1002/hsr2.71255)
Supplement: Supplementary file 1 — Supplementary Figure 1: Meta‐analysis random‐effects estimates of association between weight increase and NAFLD OR. [file HSR2-8-e71255-s001.docx]

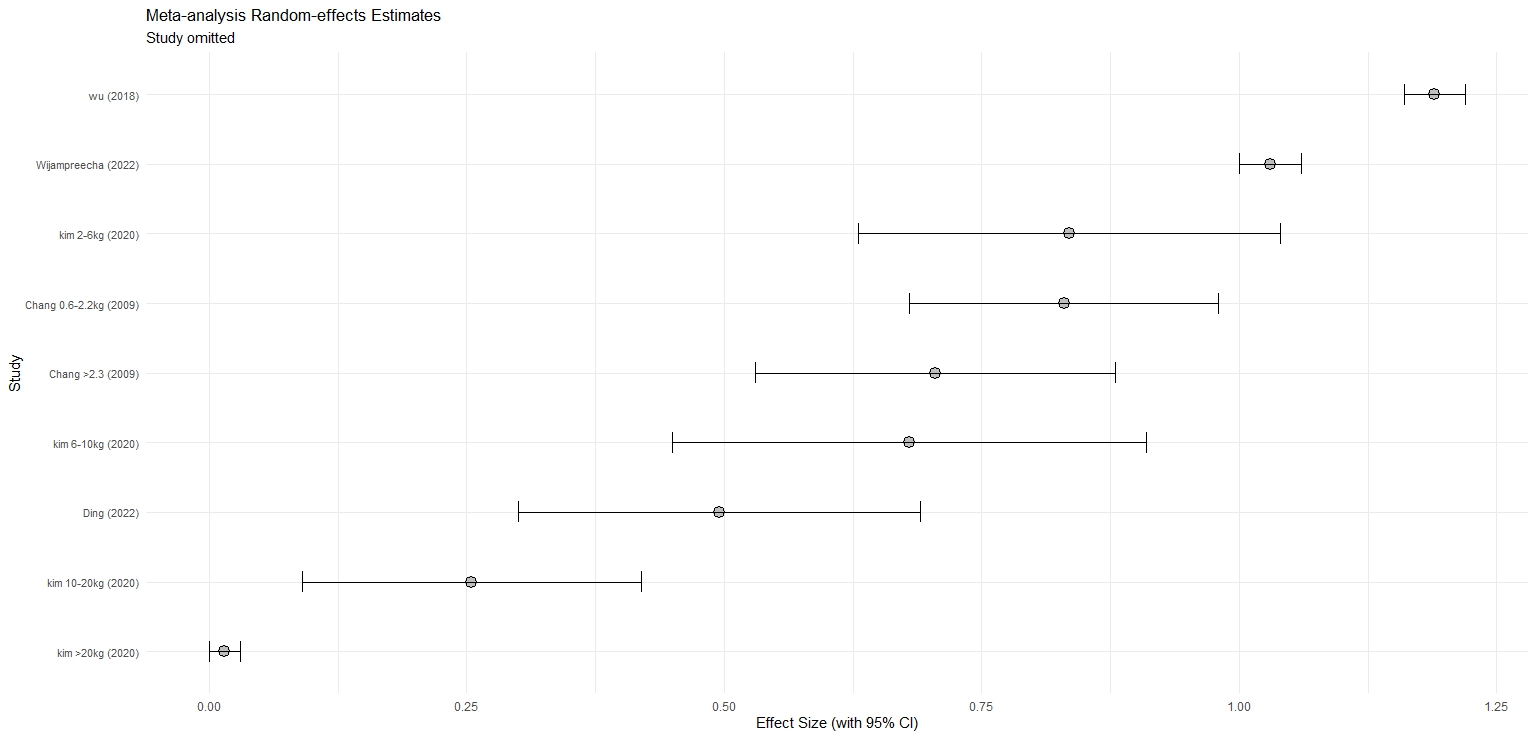


**Supplementary Figure1.** Meta-analysis random-effects estimates of association between weight increase and NAFLD OR.
